# Supplementary material for: Circulation of respiratory viruses during the COVID-19 pandemic in The Gambia
Source: Gates Open Res. 2023 Mar 27;6:148. Originally published 2022 Dec 8. [Version 3] doi: 10.12688/gatesopenres.14155.3 (PMC9883272; doi:10.12688/gatesopenres.14155.3)
Supplement: Peer review history

# Peer review: Reviewer response for version 1

**DOI:** 10.21956/gatesopenres.15460.r32799

**Published:** 2023-01-27

**Authors:** Antonia Ho

**Affiliations:** 1 Medical Research Council-University of Glasgow Centre for Virus Research, University of Glasgow, Glasgow, UK

For transparency, I have reviewed this manuscript for a different journal in September 2022. I note the authors have incorporated most of my suggested edits. This is therefore an update of the original review.

 This study reports the prevalence of respiratory viruses across different age groups from nasopharyngeal swabs collected by 2 studies in The Gambia: i) ILI surveillance at an urban and a rural outpatient clinic; ii) a clinical trial of COVID-19 treatment based in urban and peri-urban areas. Existing studies that have evaluated respiratory viral circulation during the COVID-19 pandemic have predominantly been conducted in temperate settings; the majority have shown substantial reduction in activity of most respiratory viruses, which have been attributed to the strict implementation of non-pharmacological interventions.

 Data on the circulation of respiratory viruses other than SARS-CoV-2 outside of high-income, temperate settings are scarce. Hence, data presented in this study are important. Similar to many African countries, Gambia imposed NPIs during the first pandemic wave (June to September 2020), but have not enforced NPIs since. Limited pre-pandemic data show that influenza circulated during the rainy season in the Gambia.

 The authors identified high virus positivity (44.2%) among individuals presenting with ILI between April 2020-March 2022, especially in children <5 years (80%), compared to older age groups (39.5% in 18-50s & 39.9% in >50 years). SARS-CoV-2 was the most frequently detected virus, with higher prevalence in adult age groups, while rhinovirus, RSV and hMPV were more frequently detected in children. Influenza A circulated in September to December in 2020 and 2021, asynchronously to SARS-CoV-2, while influenza B was only identified in 2020. Another interesting finding is that peaks in rhinovirus activity were also asynchronous to SARS-CoV-2 waves, mirroring recent observations from laboratory studies.

 The manuscript is well written, and contains important data. It has a number of limitations, i.e. limited demographic and clinical data, differing recruitment periods and eligibility criteria. The latter points are acknowledged by the authors.

 Comments:

1. Local SARS-CoV-2 and influenza vaccination policy should be stated in the Methods.
2. More detail on the PaTS trial should be included. It is an RCT comparing Ivermectin vs. aspirin vs. placebo. Were samples included from participants from both cohorts 1 and 2? 
3. The sampling strategy of ILI surveillance (study 1) should be included; age criteria, target numbers per day/week, as number of included samples seems to vary greatly according to Figure 2.
4. Limited demographic/clinical data are included, presumably not collected by ILI surveillance. Co-morbidities and vaccination status would have been useful.
5. The manuscript highlights scarce data on respiratory viral circulation from Africa - and mentions data from South Africa (Tempia Eurosurveillance 2021) and Ntagereka (Infect Dis med Micro 2022), but should also include Loevinsohn *et al.* (2022 ^1^), which was published prior to this manuscript. This surveillance study in Zambia found a substantial decline in the prevalence of RSV and influenza in the first year of the pandemic, while an increase in the prevalence of rhino/enteroviruses and non-SARS-CoV-2 coronaviruses was observed after March 2020 (when SARS-CoV-2 emerged in Zambia), compared to 2019. 

Is the work clearly and accurately presented and does it cite the current literature?

Yes

If applicable, is the statistical analysis and its interpretation appropriate?

Yes

Are all the source data underlying the results available to ensure full reproducibility?

Yes

Is the study design appropriate and is the work technically sound?

Yes

Are the conclusions drawn adequately supported by the results?

Yes

Are sufficient details of methods and analysis provided to allow replication by others?

Partly

Reviewer Expertise:

Respiratory viral epidemiology.

I confirm that I have read this submission and believe that I have an appropriate level of expertise to confirm that it is of an acceptable scientific standard.

## References

[1] : Respiratory viruses in rural Zambia before and during the COVID-19 pandemic. *Trop Med Int Health* .2022;27(7) : 10.1111/tmi.13781 647-654 10.1111/tmi.13781 35611546PMC9348166

# Response

**Published:** 2023-02-08

**Authors:** Sheikh Jarju

**Affiliations:** Medical Research Council (UK) The Gambia, The Gambia

**Comments:**

**1. Local SARS-CoV-2 and influenza vaccination policy should be stated in the Methods.**

 We agree that it is important to highlight the local SARS-CoV-2 and influenza vaccination policy. In the Gambia Government national deployment and vaccination plan for COVID-19 vaccines, priority groups are highlighted that includes health personnel, community workers, older people of age 65 years and above and people with pre-existing conditions would be considered for vaccination first, in that order ( **The Gambia Government, 2020; The Republic of the Gambia national deployment and vaccination plan for COVID-19; Banjul**). We have now referenced this document.

 However, there is no influenza vaccine policy in The Gambia, which we state in the methods.

**More detail on the PaTS trial should be included. It is an RCT comparing Ivermectin vs.**

**aspirin vs. placebo. Were samples included from participants from both cohorts 1 and 2?**

 The samples from PaTS included in this study were those initially screened to qualify for subsequent recruitment into cohort 1 or 2. This was at the initial stage of the study. Following successful screening and 2 ^nd^ consenting to join the study, samples were then followed up and as per severity of conditions, they were then distributed into cohort 1 or 2.  We only stopped at the screening level and didn’t include samples that were already in cohort 1 or 2. We have tried to make this clearer in the methods.

 2. **The sampling strategy of ILI surveillance (study 1) should be included; age criteria, target**

**numbers per day/week, as number of included samples seems to vary greatly according to**

**Figure 2.**

 The ILI surveillance (study 1) recruited all age groups. We recruited all participants that fulfilled the case definition in a day/week and were willing to participate in the study. We have added some text to the methods to make this clearer. The fluctuation in numbers could be due to a number of reasons, but most likely due to the number of patients presenting with ILI.

 3. **Limited demographic/clinical data are included, presumably not collected by ILI**

**surveillance. Co-morbidities and vaccination status would have been useful.**

 We agree that these aspects would be interesting. Unfortunately, we do not have extensive and systematically collected data on these variables from all the various data sources included in this ILI study.

 4. **The manuscript highlights scarce data on respiratory viral circulation from Africa - and** **mentions data from South Africa (Tempia Eurosurveillance 2021) and Ntagereka (Infect Dis** **med Micro 2022), but should also include Loevinsohn et al. (20221), which was published** **prior to this manuscript. This surveillance study in Zambia found a substantial decline in the** **prevalence of RSV and influenza in the first year of the pandemic, while an increase in the** **prevalence of rhino/enteroviruses and non-SARS-CoV-2 coronaviruses was observed after** **March 2020 (when SARS-CoV-2 emerged in Zambia), compared to 2019.**

 We agree that the Zambia paper is an important publication to reference in our manuscript and have discussed this paper.

# Peer review: Reviewer response for version 1

**DOI:** 10.21956/gatesopenres.15460.r32800

**Published:** 2023-01-18

**Authors:** Ivy A. Asante

**Affiliations:** 1 Noguchi Memorial Institute for Medical Research, University of Ghana, Accra, Ghana

In this paper, the authors describe their observations concerning respiratory pathogens during the COVID-19 pandemic in The Gambia. The data they describe is very important especially from West Africa where such data is not readily available.

 Comments:

1. From Figure 1, it looks like the authors looked at some selected sites in The Gambia, as opposed to the whole country. Even if sites were few and distributed throughout the country, it would be said to represent the country. For consideration, the authors could include ‘selected sites in The Gambia’ in their title.
2. The authors used a multiplex RT-PCR assay that detected a panel of respiratory pathogens. This detection however did not distinguish or sub-type viruses seen. E.g. for influenza B, lineage determination was not reported, same for influenza A and the human coronaviruses. If this data is available, it should be included.
3. Specify what UTM was used. What is the volume of the Universal transport medium? Why did the researchers choose to collect only NP swabs and not a combined OP/NP swab?
4. For the ILI surveillance system, is there a systematic approach to sample collection? E.g. A specific number expected each week? This could explain the number of samples tested during each of the months; what accounted for the high number of samples in one month and a low number in another?
5. Is the SARS-CoV-2 data observed in this study comparable to national data? It is not very clear.
6. Was there any correlation between detection of SARS-CoV-2 and other respiratory viruses?

Is the work clearly and accurately presented and does it cite the current literature?

Yes

If applicable, is the statistical analysis and its interpretation appropriate?

Yes

Are all the source data underlying the results available to ensure full reproducibility?

Yes

Is the study design appropriate and is the work technically sound?

Yes

Are the conclusions drawn adequately supported by the results?

Yes

Are sufficient details of methods and analysis provided to allow replication by others?

Partly

Reviewer Expertise:

Virology, surveillance for respiratory viruses

I confirm that I have read this submission and believe that I have an appropriate level of expertise to confirm that it is of an acceptable scientific standard.

# Response

**Published:** 2023-02-08

**Authors:** Sheikh Jarju

**Affiliations:** Medical Research Council (UK) The Gambia, The Gambia

Comments:

**1. From Figure 1, it looks like the authors looked at some selected sites in The Gambia, as** **opposed to the whole country. Even if sites were few and distributed throughout the** **country, it would be said to represent the country. For consideration, the authors could** **include ‘selected sites in The Gambia’ in their title.**

 We appreciate the reviewer’s point that the study only included data from limited sites. The sites selected represented both an urban and rural settlement of the country and many people seek medical care in the urban clinics we have included from different part of the country. We have discussed these limitations but feel that we do not need to change the title of the manuscript.

**2. The authors used a multiplex RT-PCR assay that detected a panel of respiratory pathogens. This detection however did not distinguish, or sub-type viruses seen. E.g., for influenza B, lineage determination was not reported, same for influenza A and the human.**

**coronaviruses. If this data is available, it should be included.**

 We agree with the suggestion that lineage information, for example influenza virus, would have added an additional information. Unfortunately, we do not have this data as all seasonal coronaviruses were detected on one probe. We do, however, have a specific H1N1 assay and what we report are influenza A data stratified by whether they were positive in the influenza A assay alone (which would pick up both H3N2 and H1N1) or in both this assay and the H1N1 specific assay. This is stated in the table legends.

**3. Specify what UTM was used. What is the volume of the Universal transport medium? Why**

**did the researchers choose to collect only NP swabs and not a combined OP/NP swab?**

 We did use UTM Copan 360C with 1ml medium. This has been added to the methods. We used nasopharyngeal swabs as the method of choice due to the extensive experience in our field teams in NPS collection during many studies of viral and bacterial carriage.

**4. or the ILI surveillance system, is there a systematic approach to sample collection? E.g. A specific number expected each week. This could explain the number of samples tested during each of the months; what accounted for the high number of samples in one month and a low number in another?**

 We have included a statement about this in response to a comment from reviewer 1 in the methods. We attempted to recruit everyone who fulfilled the case definition and was willing to participate in the study.

**5. Is the SARS-CoV-2 data observed in this study comparable to national data? It is not very** **clear.**

 Yes, the SARS-CoV-2 data observed in our study is comparable with the national data. We have stated this and added a reference.

**6. Was there any correlation between detection of SARS-CoV-2 and other respiratory viruses?**

 No clear correlations are present. We have reported the number of co-infections and also highlighted the potential viral interference observed between SARS-CoV-2, influenza and human rhinoviruses in terms of the peaks observed. This is included in the discussion already.
